# Feeding citrus pomace fermented with combined probiotics improves growth performance, meat quality, fatty acid profile, and antioxidant capacity in yellow-feathered broilers

**DOI:** 10.3389/fvets.2024.1469947

**Published:** 2024-12-20

**Authors:** Yanchen Liu, Yantian Tang, Huadi Mei, Zhichang Liu, Zhenming Li, Xianyong Ma, Zhihui Luo, Weiwen Huang, Yuanfei Li, Miao Yu

**Affiliations:** ^1^State Key Laboratory of Swine and Poultry Breeding Industry, Key Laboratory of Animal Nutrition and Feed Science in South China, Ministry of Agriculture and Rural Affairs, Guangdong Provincial Key Laboratory of Animal Breeding and Nutrition, Guangdong Engineering Technology Research Center of Animal Meat Quality and Safety Control and Evaluation, Institute of Animal Science, Guangdong Academy of Agricultural Sciences, Guangzhou, China; ^2^Guangdong Laboratory for Lingnan Modern Agriculture, Heyuan Branch, Heyuan, China; ^3^Longping Huangmang Ecological Agriculture Farm, Qingyuan, China; ^4^Kaiping Xufeng Farming and Husbandry Co., Ltd, Jiangmen, China

**Keywords:** fermented citrus pomace, yellow-feathered broilers, growth performance, meat quality, antioxidant capacity, fatty acid composition, lipid metabolism

## Abstract

**Introduction:**

The reasonable and efficient utilization of agricultural by-products as animal feed has the capacity to not only mitigate the scarcity of conventional feedstuff but also alleviate the environmental load. This study was aimed to investigate the effects of feeding citrus pomace (CP) fermented with combined probiotics on growth performance, carcass traits, meat quality and antioxidant capacity in yellow-feathered broilers.

**Methods:**

A cohort of 540 female yellow-feathered broilers (Qingyuan partridge chicken, 90-day-old) were randomly divided into three groups and, respectively, fed the basal diet (Control), diet containing 10% unfermented CP (UFCP) and diet containing 10% fermented CP (FCP).

**Results:**

The results showed that dietary FCP significantly increased (*p* < 0.05) the final-body-weight and average-daily-gain of broilers, and the pH_45 min_ and b*_24 h_ values in breast muscle, while tendentiously lowering the feed-to-gain ratio (*p* = 0.076). The levels of inosine monophosphate (*p* < 0.05) and intramuscular fat (*p* = 0.083) in the FCP group were higher than those in the control group. Remarkably, dietary FCP and UFCP increased the levels of polyunsaturated fatty acids (PUFAs) and n-6 PUFAs (*p* < 0.05). Moreover, dietary FCP decreased (*p* < 0.05) the malondialdehyde content and increased (*p* < 0.05) the glutathione peroxidase content in serum. Ingestion of FCP and UFCP increased the levels of total antioxidant capacity and catalase activity in serum, and concentrations of glutathione peroxidase and catalase in breast muscle (*p* < 0.05). Additionally, diet containing FCP or UFCP upregulated the expression of *SREBP − 1c*, *FAS*, *NRF2*, *GSH-Px*, and *CAT* in breast muscle (*p* < 0.05).

**Discussion:**

Overall, dietary supplementation with FCP obviously improved meat quality, enhanced the antioxidant capacity and regulated the lipid metabolism, contributing to the improvement of growth performance of yellow-feathered broilers.

## 1 Introduction

Citrus pomace (CP), encompassing peels, pulps, and seeds, is considered as an environmentally sustainable and economically viable agricultural by-product derived mainly from citrus processing, juicing, or canning in the citrus juice industries. It has been reported that approximately 110 to 120 million tons of CP by-products are generated per year globally (1). In addition to the abundant output, CP exhibits a comprehensive nutritional profile, featuring substantial amounts of crude protein, crude fat, and a diverse array of amino acids; moreover, it contains considerable abundant bioactive substances, such as vitamins, mineral elements, polyphenols, flavonoids, and carotenoids (2). However, owing to the dearth of rational and effective means of disposal, CP is often directly discarded or burned, leading to serious environmental pollution. The nutritional capacity and low price of CP position it as a highly promising candidate for animal feed sources, offering the potential for consumers to obtain antibiotic-free meat and egg products (3, 4).

Nowadays, microbial fermentation has garnered significant interest in the utilization of agro-industrial residues due to its capacity for large-scale treatment and enhancement of their nutritional value and functional attributes of these residues (5). It has been reported that microbial fermentation could improve the digestibility and palatability of agricultural by-products, alongside the concomitant reduction in toxic components of the by-products (5). In addition, solid-state fermentation using *Candida utilis* and *Bacillus subtilis* could remarkably increase the content of crude protein, soluble protein, and essential amino acids, while reducing the content of anti-nutritional factors (ANFs) in citrus pulp residue (6). Several studies have demonstrated that the incorporation of microbial fermented citrus peel meal as a partial substitute for corn in chicken diets can significantly enhance growth performance, reduce the feed-to-gain ratio, and exhibit potential improvements in oxidative stability of unsaturated fatty acids in meat (7–9). Additionally, a recent study has shown that supplementation of broiler diets with a methanolic extract of citrus peeling can enhance growth performance, improve immunity, and increase nutrient utilization (10). Despite this, there is currently limited research on the regulation of antioxidant capacity and meat quality in fermented citrus residue fed to broilers.

Yellow-feathered broiler, a native breed of broiler in China, is extensively raised due to its superior meat quality and unique flavor profile, which are highly favored by consumers. However, compared to the white-feathered broiler, it exhibited a slower growth rate and high feed conversion ratio (FCR), leading to elevated feeding costs (11, 12). In addition, the intensive farming of broilers, coupled with an excessive supply of energy-dense feed, has resulted in the production of meat characterized by elevated levels of saturated fat (13). Our previous studies have demonstrated that dietary supplementation with 10 mg/kg citrus extract in the diet of yellow-feather broilers can effectively enhance growth performance, exert anti-inflammatory and antioxidant effects, and improve meat quality (14). On the contrary, the utilization of citrus extract is restricted by its exorbitant cost. Citrus pomace, due to its abundant yield, cost-effectiveness, and substantial nutritional value, can potentially serve as a partial substitute for various feed components such as corn or soybean meal. In our previous *in vitro* study, results demonstrated that co-fermentation with *Aspergillus Niger*, *Candida tropicalis*, *Lactobacillus plantarum*, and *Bacillus subtilis* significantly enhanced the protein content while substantially reducing the crude fiber content of citrus residue (15).

Accordingly, we hypothesized that partial replacement of corn with FCP in the diet may have a positive impact on growth performance, meat quality, fatty acid profile and antioxidant capacity of yellow-feather broilers. To test the hypothesis, this study was conducted to investigate the effects of FCP on growth performance, carcass traits, meat quality, muscle fatty acid profile and antioxidant capacity of yellow-feather broilers. Our study aims to provide a theoretical foundation for the potential implementation of FCP in yellow-feather broiler production, and more broadly, in the broiler industry.

## 2 Materials and methods

### 2.1 Citrus pomace, fermentation strains, and preparation of fermented citrus pomace

The citrus pomace was purchased from Chongqing Tianbang Food Co., Ltd. (Chong-qing, China). The strain of *Aspergillus niger* and *Candida tropicalis* were obtained from the Sericulture and Agricultural Products Processing Institute of Guangdong Academy of Agricultural Sciences (Guangzhou, China). The strain of *Lactobacillus plantarum* and *Bacillus subtilis* were from our laboratory.

Preparation of FCP following our previously reported optimized fermentation method (15). In brief, citrus pomace with 60% moisture was inoculated with 20% inoculation amounts of complex probiotics of *Aspergillus niger*, *Candida tropicalis*, *Bacillus subtilis*, and *Lactobacillus plantarum* in the proportions of 1:1:1:1. The solid fermentation was processed at 30°C for 8 d after thorough mixing, FCP was then obtained. The routine nutrient levels of FCP and unfermented citrus pomace (UFCP) followed our previous study (15).

### 2.2 Animals, experimental design, and diets

A total of 540 ninety-day-old healthy female yellow-feathered broiler chickens (Qingyuan partridge chicken) were housed in single-layer cages (length 3.2 m × width 1.5 m × height 1.8 m) in which the floors were covered with rice husk. The broilers were raised in a sealed room with a constant temperature of 24°C and a relative humidity ranging from 50 to 60%. The light period consisted of 16 h of light and 8 h of darkness. After a 4-day adaptation period, broilers were then weighed and randomly assigned to 3 treatment groups following their body weight (BW), each group with 6 replicate cages of 30 chickens. Dietary treatment included the following: control (basal corn-soybean diet) and two other treatments in which basal diet was replaced by a 10% level of UFCP or 10% FCP (Table 1). All broilers were allowed *ad libitum* access to food and water throughout the trial period. The nutrient levels of UFCP and FCP, as measured in our previous studies (15), were utilized for the formulation of three isonitrogenous and isoenergetic diets. The diets (Table 1) among each group were formulated to meet the nutritional requirements of the National Research Council (16) and the Chinese Feeding Standard of Chicken (17). At the age at 94 or 124 days, the feed was cut off and the broilers in each cage were fasted. The weight of each broiler in cages was measured, and the average weight of each cage was calculated. Additionally, the daily feed intake for each cage was recorded. Subsequently, the average daily feed intake (ADFI), the average daily gain (ADG), and the feed to gain ratio (F/G) were calculated from 6 replicate cages per treatment. The F/G ratio was calculated as the total feed intake of six cage per group (kg)/(weight of all broilers in each group at 124 d [kg] – weight of all broilers in each group at 94 d [kg]).

**Table 1:** Feed ingredient and nutrient composition of experimental diets (as-fed basis).

Items	Treatment		
	CON	UFCP	FCP
Ingredient (%)			
Corn	70.40	60.29	60.29
Soybean meal	16.75	15.90	15.90
Wheat bran	6.00	6.00	6.00
Soybean oil	3.00	3.80	3.80
*L*-Lysine-HCl (98%)	0.19	0.25	0.25
*DL*-Methionine	0.13	0.18	0.18
*L*-Threonine	0.10	0.10	0.10
Dicalcium phosphate	1.60	1.65	1.65
Limestone	0.53	0.53	0.53
Salt	0.30	0.30	0.30
Citrus pomace	0.00	10.00	10.00
Vitamin-mineral premix^1^	1.00	1.00	1.00
Total	100.00	100.00	100.00
Nutrient levels (%)^2^			
ME^3^, MJ/kg	18.19	18.37	18.33
Crude protein	17.56	17.29	17.42
Crude ash	4.75	4.92	4.96
Crude fat	2.54	3.27	3.47
Calcium	2.61	3.10	3.23
Total phosphorus	0.40	0.35	0.35

1 Provided per kilogram of complete diet: vitamin A, 8,000 IU; vitamin D_3_, 2,800 IU; vitamin E, 19 mg; vitamin K_3_, 3.32 mg; vitamin B_1_, 1.7 mg; vitamin B_2_, 8.2 mg; vitamin B_6_, 2.78 mg; vitamin B_12_, 0.015 mg; niacin, 30 mg; calcium pantothenate, 10.9 mg; folic acid, 0.95 mg; biotin, 0.16 mg; Fe (FeSO_4_·H_2_O), 81 mg; Cu (CuSO_4_·5H_2_O), 9 mg; I (KI),0.50 mg; Se (Na_2_SeO_3_), 0.27 mg; Zn (ZnSO_4_·H_2_O), 68 mg; Mn (MnSO_4_·H_2_O), 82 mg.

2 The Nutrient levels were measured values.

3 ME, metabolizable energy.

### 2.3 Slaughtering and sampling

After 8 h of fasting on the last day of rearing, two broilers around the average BW from each replicate cage were electrically stunned and exsanguinated. Broilers were drawn using sterile syringes via the wing vein. The blood samples were centrifuged (at 3,000× rpm for 5 min) to obtain serum and then stored at −20°C for additional analyses. The left breast muscle from the same region was sampled and divided into three parts: one part was sealed in a polyethylene vacuum bag and refrigerated at 4°C for 45 min and 24 h for meat quality analysis, and the other two parts were immediately transferred into liquid nitrogen and stored at −80°C for subsequent chemical composition and quantitative real-time PCR (qRT-PCR) analyses.

### 2.4 Carcass traits and meat quality

After slaughtering, the left breast meat of 12 broilers per group were excised for measuring carcass traits and meat quality. The carcass traits data were collected and calculated as previously described (18). In brief, boilers were weighed after removing feathers, feet, and beak shells to obtain the carcass weight, and the dressing percentage, semi-eviscerated yield, eviscerated yield, breast muscle yield, leg muscle yield, and abdominal fat yield were calculated. Subsequently, the breast muscles, thigh muscles, and abdominal fat (including fat around the musculature and stomach) were divided and weighed to calculate the percentage of breast muscle, thigh muscle, and abdominal fat based on the carcass weight. At 45 min and 24 h postmortem, the pH and color (L*, a*, and b* values) of breast muscle were determined by an electronic pH meter (model HI 9024C HANNA Instruments, Ltd., Beijing, China) and Chroma meter (model CR-410 Konica Minolta Sensing Ins., Osaka, Japan) separately. The drip loss was measured as described by Zhang et al. (19). Briefly, about 3.0 cm length cube of the breast muscle was manually trimmed and weighed at 45 min postmortem, and suspended by a fishhook in an inflated plastic bag, sealed and stored for 24 h at 4°C, after which the sample was removed from the fishhook, blotted dry on filter paper, and reweighted. Drip loss was expressed as the weight change percentage of the sample before and after storage. To measure the cooking loss, about 25.0 g muscle was weighed, cooked at 85°C and cooled in running water at 4°C. The samples were wiped to remove the surface moisture and reweighed. Cooking loss was expressed as the weight change percentage of the sample before and after cooking. The shear force was determined as described by Yu et al. (20) The samples were cooked at 80°C (internal temperature at 70°C), and cooled in running water at 4°C. Then, the samples were cut to 1 cm × 1 cm × 3 cm sheared parallel to the muscle fiber direction and detected by using a C-LT3B digital-display muscle tenderness determination device (Tenovo, Harbin, China).

### 2.5 Diet and muscle chemical composition analysis

The chemical composition (metabolizable energy, crude protein, crude ash, crude fat, calcium, and total phosphorus) in experimental diets and the chemical composition (dry matter, crude protein, and IMF) in the breast muscle (12 broilers per group) were analyzed by the methods of the AOAC (21). The content of inosine monophosphate (IMP) in the breast muscle was performed with high-performance liquid chromatography (HPLC) according to the previous study (22). For the fatty acid analysis, the freeze-dried breast muscle samples were pulverized. The lipids of pulverized samples were isolated with chloroform-methanol (1,1, v/v) following Folch et al. (23). The determination of fatty acid methyl esters was carried out on an Agilent 7890B gas chromatographer system with a flame ionization detector (Agilent Technologies Inc., Santa Clara, CA, USA). The composition of fatty acids in the breast muscle was determined as previously described (24).

### 2.6 Measurement of serum and muscle antioxidant

Serum samples and muscle samples (12 broilers per group) were preprocessed according to the kit instructions. The total antioxidant capacity (T-AOC), total superoxide dismutase (T-SOD), catalase (CAT), and glutathione peroxidase (GSH-Px) activities and the malondialdehyde (MDA) content in serum and muscle were measured using specific kits (Nanjing Jiancheng Bioengineering Institute, Nanjing, China) according to the manufacturer’s instructions.

### 2.7 Total RNA extraction and quantitative real-time PCR analysis

Total RNA isolation, cDNA synthesis, and qRT-PCR were performed as the previous study (20). Primers used for selected genes are presented in Supplementary Table S1. The housekeeping gene *β*-actin was used to normalize each target gene expression level, and the relative expression levels of target gene were quantified by the 2^−∆∆Ct^ method (25).

### 2.8 Statistical analysis

All experimental data was analyzed by the one-way ANOVA by using the SPSS software package (SPSS v. 20.0, SPSS Inc., Chicago IL, United States). The growth performance was evaluated using replicate as the experimental unit, while other experimental data were evaluated using the averages of the 2 sampled broilers per replicate. Duncan’s multiple range test was utilized for differences among groups. Data are expressed as the mean ± SEM. Statistical significance was set at *p <* 0.05 and statistical trends at 0.05 < *p <* 0.10. Means within a row with different superscript letters are significantly different (*p* < 0.05).

## 3 Results

### 3.1 Growth performance and carcass traits

The growth performance and carcass traits of yellow-feathered broilers fed diets with UFCP and FCP are presented in Table 2. The broilers fed with FCP significantly increased the final BW and ADG (*p* < 0.05), and tended to decrease the F/G (*p* = 0.076) compared with the broilers fed with the basal diet and UFCP, with insignificant differences in the initial BW and ADFI among all three groups (*p* > 0.10). Additionally, dietary inclusion of FCP and UFCP did not significantly affect the carcass traits, including dressed percentage, semi-eviscerated yield, eviscerated yield, thigh muscle yield, breast muscle yield, and abdominal fat yield (*p* > 0.10).

**Table 2:** Effects of different type of citrus pomace on the growth performance and carcass traits of Yellow-feathered broiler chickens.

Items	Treatment			*p* value
	CON	UFCP	FCP	
Growth performance				
Initial BW (g)	1271.94 ± 0.28	1273.33 ± 0.61	1272.78 ± 0.56	0.178
Final BW (g)	1768 ± 7.76^b^	1781.83 ± 15.93^b^	1814.33 ± 2.25^a^	0.018
ADFI (g/d)	89.10 ± 2.54	91.46 ± 0.85	88.41 ± 1.65	0.479
ADG (g/d)	17.11 ± 0.27^b^	17.53 ± 0.55^b^	18.67 ± 0.09^a^	0.020
F/G	5.22 ± 0.21	5.24 ± 0.17	4.74 ± 0.10	0.076
Carcass traits (%)				
Dressed percentage	90.63 ± 0.57	90.14 ± 0.59	89.68 ± 0.89	0.639
Semi-eviscerated yield	79.19 ± 0.97	78.61 ± 0.92	79.74 ± 0.61	0.649
Eviscerated yield	71.30 ± 0.54	70.65 ± 0.97	70.80 ± 0.95	0.848
Thigh muscle yield	11.15 ± 0.48	10.74 ± 0.32	11.10 ± 0.44	0.744
Breast muscle yield	6.33 ± 0.17	5.80 ± 0.14	6.89 ± 0.98	0.440
Abdominal fat yield	4.35 ± 0.32	4.74 ± 0.47	4.59 ± 0.63	0.855

Data were shown as Mean ± SEM (*n* = 12). Means within a row with different superscript letters are significantly different (*p* < 0.05). BW, body weight; ADG, average daily gain; ADFI, average daily feed intake; F/G, feed to gain ratio.

### 3.2 Meat quality and muscle chemical compositions

The meat quality and muscle chemical composition of broilers is shown in Table 3. The broilers fed with FCP significantly increased the pH_45 min_, b^*^_24 h_, and IMP content in the breast muscle (*p* < 0.05), and tended to increase the IMF content (*p* = 0.083). The pH_45 min_ and IMP content of breast muscle were increased but were insignificant in broilers treated with UFCP (*p* > 0.10). In addition, there was no significant difference in pH_24 h_, L^*^(45 min and 24 h), a^*^(45 min and 24 h), b^*^_45 min_, drip loss, cooking loss, shear force, and DM and CP contents among the three groups (*p* > 0.10).

**Table 3:** Effects of different type of citrus pomace on the meat quality and muscle chemical compositions of Yellow-feathered broiler chickens.

Items	Treatment			*p* value
	CON	UFCP	FCP	
pH_45 min_	5.83 ± 0.04^b^	6.03 ± 0.08^ab^	6.11 ± 0.08^a^	0.034
pH_24 h_	5.80 ± 0.03	5.81 ± 0.03	5.77 ± 0.02	0.524
L^*^_45 min_	58.45 ± 1.30	57.53 ± 0.79	59.36 ± 0.55	0.404
L^*^_24 h_	60.84 ± 0.90	61.06 ± 0.42	60.05 ± 1.53	0.781
a^*^_45 min_	13.47 ± 0.53	13.60 ± 0.44	12.17 ± 0.51	0.111
a^*^_24 h_	12.03 ± 0.23	12.41 ± 0.20	11.65 ± 0.35	0.168
b^*^_45 min_	13.83 ± 0.60	12.54 ± 0.32	13.48 ± 0.48	0.177
b^*^_24 h_	13.79 ± 0.39^b^	13.59 ± 0.45^b^	16.34 ± 1.00^a^	0.019
Drip loss (%)	3.53 ± 0.31	2.87 ± 0.27	2.80 ± 0.20	0.142
Cooking loss (%)	8.17 ± 0.31	8.03 ± 0.52	8.70 ± 0.69	0.654
Shear force (N)	27.93 ± 2.15	25.20 ± 1.73	26.49 ± 1.75	0.601
Chemical compositions				
DM (%)	49.42 ± 1.34	52.49 ± 1.32	51.28 ± 1.92	0.382
CP (%)	90.70 ± 0.85	90.85 ± 0.10	90.77 ± 0.32	0.986
IMF (%)	1.46 ± 0.10	1.42 ± 0.15	1.90 ± 0.20	0.083
IMP (mg/g)	228.50 ± 5.37^b^	246.67 ± 11.44^ab^	262.17 ± 5.99^a^	0.032

Data were shown as Mean ± SEM (*n* = 12). Means within a row with different superscript letters are significantly different (*p* < 0.05). DM, dry matter; CP, crude protein; IMF, intramuscular fat; IMP, inosine monophosphate.

### 3.3 Fatty acid profiles

The fatty acid profiles of the breast muscle are presented in Table 4. The broilers fed with FCP and UFCP increased the *Σ* PUFA and Σ n-6 PUFA contents (*p* < 0.05), and tended to increase the Σ n-3 PUFA content (*p* = 0.061) and decrease the C18:1n9 (*p* = 0.056) as well as *Σ* monounsaturated fatty acids (MUFA) (*p* = 0.057) contents. In addition, the content of C18:2n6 in the breast muscle was significantly increased in broilers fed with UFCP (*p* < 0.05). The content of C18:2n6 was increased by dietary inclusion of FCP, but not significant (*p* > 0.10). Moreover, results showed that FCP and UFCP had no effects on the contents of C14:0, C16:0, C16:1n7, C18:0, C18:3n3, C20:3n6, C20:4n6, C24:0, C22:6n3, *Σ* saturated fatty acids (SFA) and Σ unsaturated fatty acids (UFA), and the ratio of Σ n-6/n-3 PUFA (*p* > 0.10).

**Table 4:** Effects of different type of citrus pomace on fatty acid profile of breast muscle of Yellow-feathered broiler chickens (% of total fatty acids).

Items	Treatment			*p* value
	CON	UFCP	FCP	
C14:0	0.56 ± 0.02	0.54 ± 0.03	0.56 ± 0.01	0.856
C16:0	24.45 ± 0.21	23.21 ± 0.56	24.00 ± 0.27	0.094
C16:1n7	3.45 ± 0.23	2.54 ± 0.41	2.79 ± 0.40	0.208
C18:0	7.81 ± 0.29	8.86 ± 0.53	8.83 ± 0.71	0.319
C18:1n9	32.81 ± 1.07	28.19 ± 1.28	29.58 ± 1.43	0.056
C18:2n6	23.55 ± 0.28^b^	26.54 ± 0.82^a^	25.07 ± 0.31^ab^	0.004
C18:3n3	1.43 ± 0.05	1.73 ± 0.29	1.51 ± 0.04	0.450
C20:3n6	0.41 ± 0.08	0.63 ± 0.11	0.54 ± 0.08	0.274
C20:4n6	3.66 ± 0.72	5.60 ± 0.85	4.95 ± 0.82	0.249
C24:0	0.76 ± 0.13	1.24 ± 0.20	0.97 ± 0.15	0.158
C22:6n3	0.52 ± 0.12	0.89 ± 0.17	0.76 ± 0.17	0.251
*Σ* SFA	33.59 ± 0.46	33.76 ± 0.95	34.37 ± 1.07	0.804
*Σ* UFA	65.76 ± 0.26	66.01 ± 0.82	65.20 ± 0.90	0.721
Σ MUFA	36.26 ± 1.16	30.72 ± 1.60	32.36 ± 1.75	0.057
Σ PUFA	29.50 ± 0.94^b^	35.29 ± 1.25^a^	32.84 ± 0.90^a^	0.005
Σ n-6 PUFA	27.55 ± 0.87^b^	32.66 ± 1.23^a^	30.57 ± 0.80^a^	0.008
Σ n-3 PUFA	1.95 ± 0.09	2.63 ± 0.27	2.27 ± 0.13	0.061
Σ n-6/n-3 PUFA	14.20 ± 0.45	13.01 ± 1.24	13.61 ± 0.57	0.607

Data were shown as Mean ± SEM (*n* = 12). Means within a row with different superscript letters are significantly different (*p* < 0.05). Σ SFA, saturated fatty acids (sum of C14:0, C16:0, C18:0, and C24:0); Σ MUFA, monounsaturated fatty acids (sum of C16:1n7 and C18:1n9); Σ PUFA, polyunsaturated fatty acids (sum of C18:2n6, C18:3n3, C20:3n6, C20:4n6, and C22:6n3); Σ UFA, unsaturated fatty acids (sum of MUFA and PUFA).

### 3.4 Antioxidant capacity

The antioxidant capacity in serum and breast muscle is shown in Table 5. Dietary supplementation with FCP and UFCP considerably increased the serum level of T-AOC, bolstered the activity of CAT in serum, and the activity of CAT and GSH-Px in breast muscle (*p* < 0.05). Additionally, the incorporation of FCP into the diets remarkably reduced the content of MDA and increased the GSH-Px activity in serum, respectively (*p* < 0.05).

**Table 5:** Effects of different type of citrus pomace on antioxidant capacity of of Yellow-feathered broiler chickens.

Items	Treatment			*p* value
	CON	UFCP	FCP	
Serum				
MDA (nmol/mL)	1.97 ± 0.23^a^	1.35 ± 0.20^ab^	1.06 ± 0.25^b^	0.036
T-AOC (mmol/L)	5.17 ± 0.41^b^	6.95 ± 0.62^a^	7.36 ± 0.23^a^	0.009
T-SOD (U/mL)	542.37 ± 47.13	622.91 ± 41.02	660.72 ± 19.59	0.111
GSH-Px (U/mL)	2797.99 ± 44.71^b^	2900.34 ± 53.99^b^	3270.44 ± 74.98^a^	<0.001
CAT (U/mL)	3.22 ± 0.32^b^	4.35 ± 0.28^a^	4.88 ± 0.39^a^	0.009
Breast muscle				
MDA (nmol/mg prot)	0.04 ± 0.01	0.04 ± 0.01	0.03 ± 0.01	0.471
T-AOC (mmol/g prot)	0.07 ± 0.01	0.06 ± 0.01	0.07 ± 0.01	0.351
T-SOD (U/mg prot)	2.82 ± 0.09	3.09 ± 0.06	2.93 ± 0.12	0.169
GSH-Px (U/mg prot)	3.48 ± 0.35^b^	4.99 ± 0.33^a^	5.03 ± 0.43^a^	0.014
CAT (U/mg prot)	3.63 ± 0.21^b^	4.47 ± 0.18^a^	4.97 ± 0.23^a^	0.001

Data were shown as Mean ± SEM (*n* = 12). Means within a row with different superscript letters are significantly different (*p* < 0.05). CAT, catalase; GSH-Px, glutathione peroxidase; MDA, malondialdehyde; SOD, superoxide dismutase; T-AOC, total antioxidant capacity.

### 3.5 Relative mRNA expression of genes associated with lipid metabolism and antioxidant capacity in breast muscle

**Figure 1:**
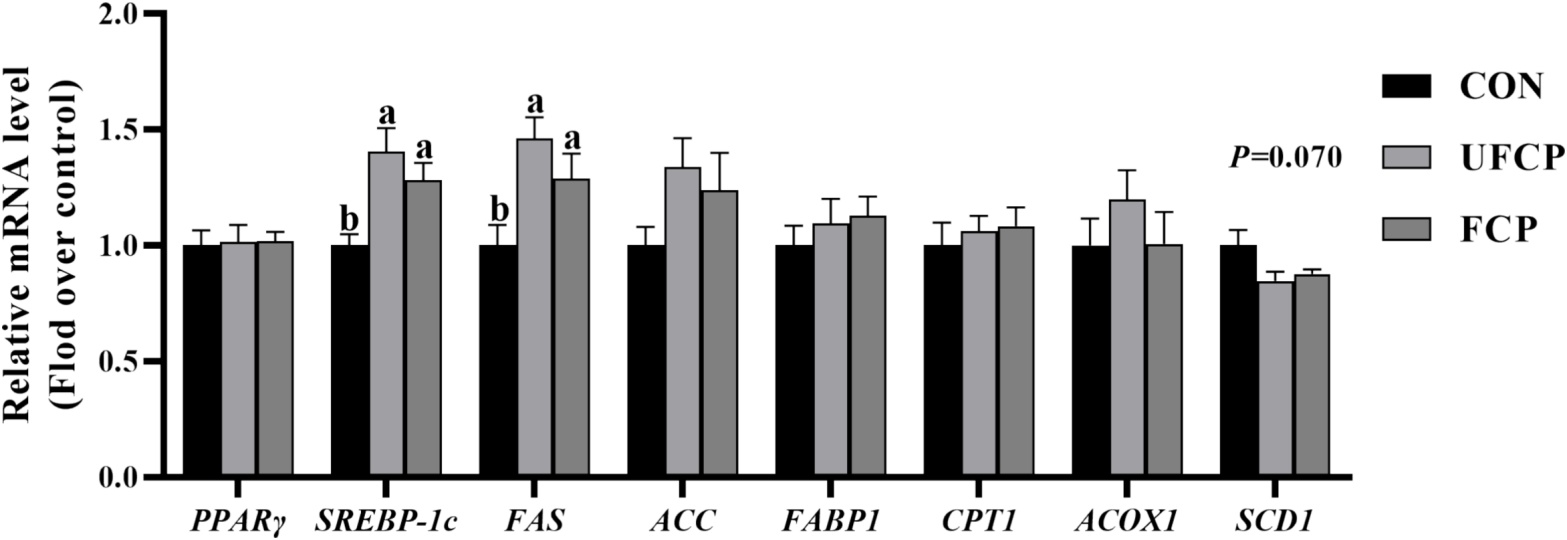
Effect of different type of citrus pomace on the mRNA expression levels of lipid metabolism in breast muscle of Yellow-feathered broiler chickens. Data were shown as Mean ± SEM (*n* = 12). Means within a row with different superscript letters are significantly different (*p* < 0.05). *PPARγ*, proliferator-activated receptors; *SREBP–1c*, Sterol regulatory element-binding protein 1c; FAS, fatty acid synthase; FABP1, Fatty Acid Binding Protein 1; CPT1, carnitine palmitoyltransferase 1; ACOX1, Acyl-CoA Oxidase 1; SCD1, Stearoyl-CoA Desaturase.

To further investigate the mechanisms involved in the regulation of IMF and PUFA deposition, the relative mRNA expression of genes related to lipid metabolism in breast muscle (Figure 1) was detected by qRT-PCR. The relative mRNA expression of *SREBP-1c* and *FAS* in the breast muscle was elevated when either FCP or UFCP was incorporated into the diets (*p* < 0.05). In addition, dietary incorporation of FCP and UFCP was tended to decreased the relative mRNA expression of *SCD1* in breast muscle (*p* = 0.070). No significant differences in the relative mRNA expression of *PPARγ*, *ACC*, *FABP1*, *CPT1*, and *ACOX1* among the three groups (*p* > 0.10).

**Figure 2:**
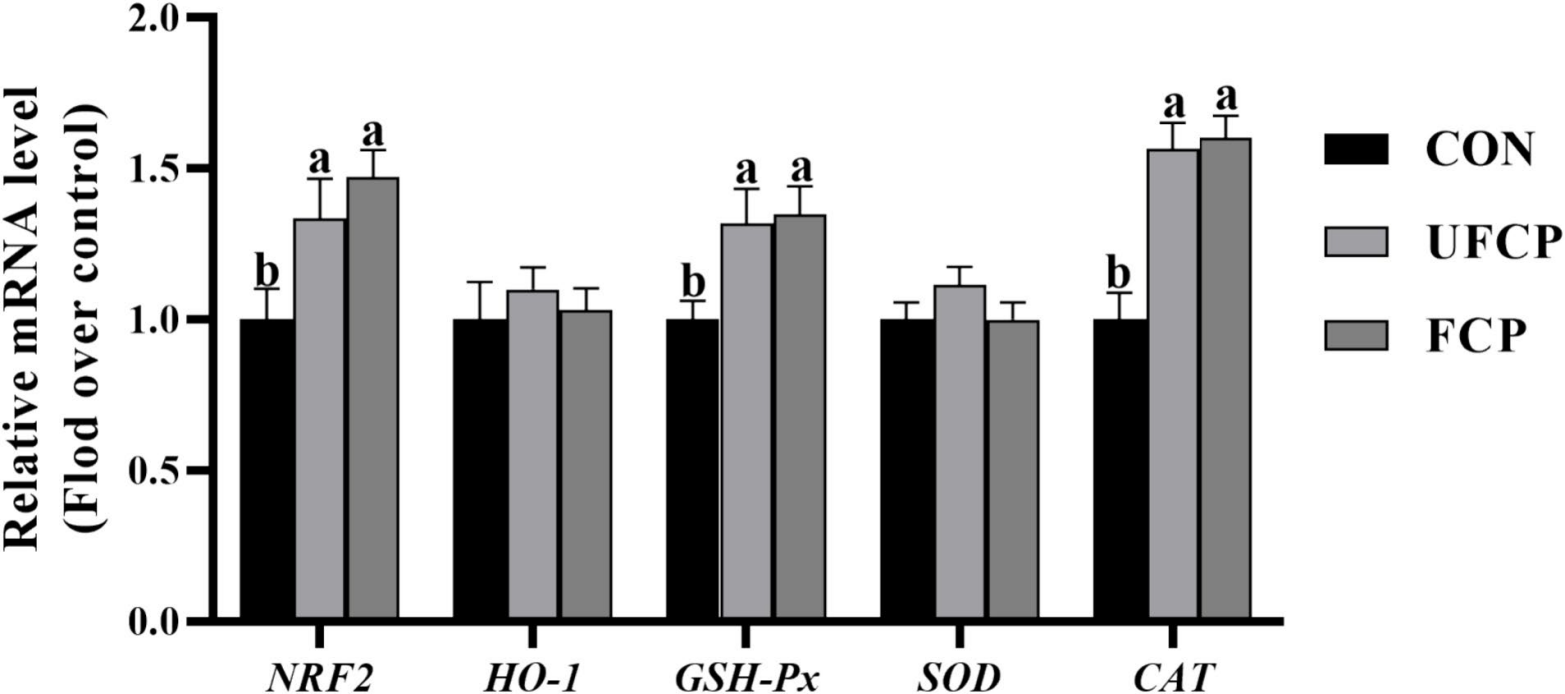
Effect of different type of citrus pomace on the mRNA expression levels of antioxidants in breast muscle of Yellow-feathered broiler chickens. Data were shown as Mean ± SEM (*n* = 12). Means within a row with different superscript letters are significantly different (*p* < 0.05). *NRF2*, nuclear factor-erythroid 2 related factor 2; HO–1, Heme Oxygenase-1; *GSH–Px*, glutathione peroxidase; SOD, Superoxide Dismutase; CAT, catalase.

The relative mRNA expression of genes associated with antioxidants in breast muscle is present in Figure 2. The relative mRNA expression of nuclear factor erythroid 2–related factor 2 (*NRF2*) and its downstream genes, including *GSH-Px* and *CAT*, were sharply upregulated following the inclusion of FCP or UFCP in the diets (*p* < 0.05). However, the incorporation of neither FCP nor UFCP did not remarkably change the relative mRNA expression of *HO-1* and *SOD* (*p* > 0.10).

## 4 Discussion

Citrus pomace, an agricultural by-product, has excellent potential for feed development in animal production due to its rich functional and nutritional components, as well as the characteristics of high yield, cost-effectiveness, and renewability (26). Unfortunately, its broad application is limited due to the presence of high fiber content and low protein content. Microbial fermentation is an effective approach that generates biological substances with health-promoting properties and ameliorates the nutritional value of plants (27). Our results showed that FCP increased the growth performance and meat quality in yellow-feathered broiler chickens. Additionally, both FCP and UFCP could improve fatty acid profile and antioxidant capacity. To sum up, FCP can be used as a good substitute for feed ingredients to improve the growth performance and antioxidant capacity of broilers and produce chicken meat that is more nutritious and meets consumer needs.

### 4.1 Effect of dietary FCP on growth performance of yellow-feather broilers

Previous studies have been carried out on the dietary inclusion of citrus pulp and citrus peel in poultry, but few studies are hitherto available on the dietary effect of citrus pomace. The growth performance can provide essential information for the application of citrus pomace in animal production. Herbal products with considerable antioxidative substances could ameliorate growth performance by enhancing antioxidant ability (28). In the current study, dietary inclusion of FCP at 10% enhanced the antioxidant capacity, increased BW and ADG, and decreased F/G in broilers. Similarly, Maha et al. (29) showed that dietary inclusion of orange peel increased the serum GSH level and improved the BW in broilers, suggesting that the effect on growth performance of broilers might be attributed to the antioxidant activity exhibited by orange peel. Nevertheless, dietary 10% UFCP supplementation did not alter the growth performance of broilers. Due to the isonitrogenous and isoenergetic diets employed in this study, inconsistent results regarding the growth performance of broilers in response to FCP and UFCP treatments may be attributed to the reduction of anti-nutritional factors (ANFs), such as non-starch polysaccharides (NSP), in citrus pomace after microbial fermentation. Citrus pomace contains many NSPs, which may have a negative impact on the digestion and absorption of nutrients (30). Due to the presence of considerable levels of NSPs in UFCP, it may have offset somewhat of the positive effect of enhanced antioxidant capacity on growth performance. The NSP in citrus pomace could be broken down during the process of microbial fermentation by generating diverse NSP enzymes (31, 32), which may assist in improving the availability and absorption of nutrients, thereby improving growth performance. Furthermore, the utilization of FCP offered several advantages in maintaining intestinal health, including the enhancement of intestinal microbial structure and the production of a greater quantity of beneficial bioactive metabolites. Consequently, it may potentially contribute to the promotion of broiler growth (33). These may partly account for the improvement in the growth performance of broilers by dietary FCP. However, further studies are necessary to investigate the exact mechanism by which FCP boosts growth due to its complex composition. These results indicated that FCP could be used as a good feed ingredient since replacing partially basal diets with FCP possesses a superior growth performance in yellow-feather broilers when compared with UFCP.

### 4.2 Effect of dietary FCP on the meat quality of yellow-feather broilers

Meat pH is an important indicator for evaluating meat quality as it is related to the shelf life, color, and water-holding capacity of meat. The value of pH was determined mainly by the degree of muscle glycolysis after animal slaughter, and a rapid pH drop can lead to protein denaturation, causing paleness and low water-holding capacity of meat, thereby reducing nutrient value (34, 35). Lu et al. (36) demonstrated that dietary 2–8% fermented pineapple residue increased the pH value at 45 min in the meat of broilers. The pineapple residue is known to contain high proportions of phenols and flavonoids, which exhibit potent antioxidant capacity (37). We obtained similar results that the pH value at 45 min in the meat of broilers was increased in both the FCP and UFCP groups. The beneficial effects of the FCP and UFCP on meat pH can be explained by polyphenols, which can reduce the activity of lactate dehydrogenase and the process and potential of anaerobic glycolysis in muscle, thereby decreasing the content of lactic acid and increasing pH value in meat (38, 39).

Meat color primarily affects consumer preferences for meat products, including L*, a*, and b* values. In this study, the b* value at 24 h of the broiler breast muscle was increased with along fed with FCP. However, dietary supplementation with UFCP was no significant in b* value at 24 h of the broiler breast muscle. Previous studies suggested that the addition to broiler feed of dried pomace of apples, cherries and strawberries showed no significant change in b* value (40). These findings suggested that the variation in b* value at 24 h of the broiler breast muscle may attributed to the retention or synthesis of specific bioactive compounds during fermentation, thereby influencing its alteration during storage. Nevertheless, further research is required to investigate the impact of FCP on variations in the b* value at 24 h in yellow-feathered broilers.

In addition, the results of this study showed that broilers fed with the FCP also increased the content of IMF and IMP in the breast muscle. IMF is one of the key indices of meat quality, positively correlated with the flavor, juiciness, and tenderness of the meat (41). IMP is considered an umami flavor enhancer, as it can affect the umami flavor of meat after cooking (20). Thus, the higher content of IMF and IMP in breast muscle indicated that FCP could partially replace corn and soybean meal in diets and may help improve meat tenderness, juiciness, and flavor. The effect of FCP and UFCP on regulating the IMF and IMP in the breast muscle of broilers has not been unambiguously investigated to date, and it is difficult to explain this phenomenon. A popular explanation for this phenomenon is that microbes and their metabolites may promote IMF accumulation and IMP synthesis in muscle (42, 43). However, there is little available information regarding the effect of FCP on meat quality, and these explanations need further verification.

The fatty acid composition of IMF serves as a vital quality attribute that consumers are concerned, it plays an important role in meat quality, and determines the flavor and nutritional value of the meat (44). Food with a higher PUFA content and a lower ratio of n-6/n-3 PUFA are readily accepted and desirable by consumers due to their nutritional value and health-enhancing properties (45, 46). Our results showed that diets supplemented with FCP and UFCP exhibit higher contents of C18:2n6, *Σ* PUFA, Σ n-6 PUFA, and Σ n-3 PUFA in breast muscle in comparison with the CON group. Similarly, Tayengwa et al. (47) found that diet supplementation with citrus pulp could increase the proportions of C18:2n6, Σ n-3 PUFA and Σ n-6 PUFA. Actually, many organisms, including but not limited to humans, cannot synthesize n-6 PUFA and n-3 PUFA by themselves, which are solely obtained from diets (48). Assefa et al. (49) reported that citrus exhibits significant antioxidant potential and is abundant in linoleic acid. As a result, the alteration of these above results might have been due to a certain level of PUFA, such as linoleic acid (C18:2n6) and linolenic acid (C18:3n3), present in citrus pomace (50, 51), they are the precursor of n-6 PUFA and n-3 PUFA families, respectively (52). Chicken meat possesses a considerable content of PUFA in comparison with other meats, so it is vulnerable to oxidative stress (35). For this reason, the use of antioxidants in the diet has been recommended to limit lipid peroxidation, maintain animal health, and achieve better product quality. Notably, studies have confirmed that plant extracts or dietary fermented blueberry pomace supplementation prevent lipoperoxidation (53). Therefore, we speculate that the increase in C18:2n6, *Σ* PUFA, Σ n-6 PUFA, and Σ n-3 PUFA in breast muscle can be partly attributed to the enhanced antioxidant properties, which could protect PUFA from oxidative stress. Indeed, enhanced antioxidant capacity in this study supports this speculation. In general, our results suggested that a diet supplemented with FCP may have a positive effect on meat quality as it contributes to increasing the values of pH and b*, the content of IMF and IMP, and improving the fatty acid composition.

### 4.3 Effect of dietary FCP on the mRNA expression levels of genes involved in the lipid metabolism of yellow-feather broilers

To investigate the molecular mechanism of IMF and PUFA changes in breast muscle, we measured the related-gene expression involved in lipid metabolism in the breast muscle, and an upregulated relative mRNA expression of lipid synthesis-related factors *SREBP1-c* and *FAS* was observed from broilers fed FCP and UFCP. SREBP1-c is a transcription factor that can regulate the expression of genes involved in the *de novo* fatty acid synthesis, including fatty acid synthesis, elongation, and desaturation (54). FAS, a well-known target of SREBP1-c, is mainly involved in the synthesis of long-chain fatty acids (55). Therefore, in this study, the IMF content in the FCP group and the proportion of PUFA, n-6 PUFA, and n-3 PUFA in FCP and UFCP groups were increased, possibly due to the upregulation of relative mRNA expression of *SREBP1-c* and *FAS*. Although dietary incorporation of UFCP upregulated the relative mRNA expression of *SREBP1-c* and *FAS*, it did not alter the IMF content in chicken meat. A previous study reported that the IMF content in the breast and thigh muscles of broiler chicks decreased with an increased NSP content from the wheat diet (56). Accordingly, the high content of NSPs in UFCP may be responsible for this phenomenon. Simultaneously, we also observed a reduction in the relative mRNA expression of *SCD1* in response to FCP and UFCP treatment. SCD1 is the rate-limiting enzyme that can convert SFA into MUFA (57). This may explain why the MUFA in chicken meat was decreased in broilers fed with FCP and UFCP.

### 4.4 Effect of dietary FCP on the antioxidant capacity of yellow-feather broilers

The antioxidant ability is positively correlated with a healthy body and meat quality (58). MDA, T-AOC, SOD, CAT, and GSH-PX are the primary indicators reflecting the degree of oxidative stress in the body. Our results showed that the dietary inclusion of FCP and UFCP increased the activities of the antioxidant enzymes GSH-Px and CAT in serum and breast muscle, and T-AOC in serum, whereas decreased the serum content of MDA. The study conducted by Gungor et al. (59) reported an increase in the activity of GSH-Px in the serum of broiler chicks following the dietary inclusion of fermented and unfermented grape pomace. Similarly, the dietary inclusion of fermented and unfermented pomegranate pomace has been found to decrease the MDA content in the breast muscle of broiler chickens (60). The activity of antioxidant enzymes depends in part on their expression levels of the antioxidant gene, which is controlled by *NRF2* (61). In the present study, we further observed that the gene expression levels of *NRF2*, *GSH-Px*, and *CAT* in breast muscle were elevated by both FCP and UFCP supplementation. Huang et al. (62) reported that apple polyphenols improve intestinal antioxidant capacity by activating the NRF2/Keap1 Signaling and improving the mRNA expression of related antioxidant genes. Consequently, we have reason to believe that dietary inclusion of FCP and UFCP augments the health of the host and oxidative stability of chicken meat by promoting the gene expression of enzymes related to antioxidants through activating the NRF2 signaling pathway. The presence of phenolic compounds and flavonoid compounds in citrus pomace may account for the enhancement in broilers’ antioxidant capacity when consuming FCP and UFCP. These bioactive substances supply a powerful antioxidant capacity to the organism by facilitating the antioxidant enzyme systems with a good radical scavenging ability (63). Collectively, these results suggested that supplementation of FCP and UFCP at 10% inclusion levels in the diets of broilers could bolster antioxidant capacity and thereby contribute to ameliorating the health and meat quality of chickens.

## 5 Conclusion

Collectively, our study revealed that the incorporation of FCP into the diet could improve the growth performance and meat quality in yellow-feather broilers. Additionally, the dietary inclusion of either 10% FCP or 10% UFCP exhibited a beneficial effect on the fatty acid profile and antioxidant capacity in yellow-feather broilers. Moreover, alteration of expression of genes related to lipid metabolism and antioxidants might support these benefits. These findings provide a promising outlook for FCP and UFCP as viable and nutrient-rich components for inclusion in broiler chicken feed formulations. Given these results above, the inclusion of FCP was recommended when added to the diet of broiler chickens. However, the optimum inclusion level of FCP in the diets of broiler chickens should be further investigated.

## Data Availability

The datasets presented in this study can be found in online repositories. The names of the repository/repositories and accession number(s) can be found in the article/Supplementary material.
