# Supplementary material for: Feeding citrus pomace fermented with combined probiotics improves growth performance, meat quality, fatty acid profile, and antioxidant capacity in yellow-feathered broilers
Source: Front Vet Sci. 2024 Dec 20;11:1469947. doi: 10.3389/fvets.2024.1469947 (PMC11729385; doi:10.3389/fvets.2024.1469947)
Supplement: Supplementary file 1 [file Table_1.DOCX]

Supplementary Material

**Table S1.** Primer Sequences Used for Quality Real-time PCR Assay

| **Genes** | **GenBank ID** | **Primer sequence (5′→3′)** | **Product size** |
| --- | --- | --- | --- |
| ***β-Actin*** | NM_205,518.1 | Forward: TGCTGTGTTCCCATCTATCG | 150 |
|  |  | Reverse: TTGGTGACAATACCGTGTTCA |  |
| ***SCD1*** | NP_990221.2 | Forward: CATGGGCCATTCTGTGCTT | 131 |
|  |  | Reverse: GGCCATGGAGTTTGCAATAG |  |
| ***CPT1*** | NM_001012898.1 | Forward: ACAGCGAATGAAAGCAGGGT | 93 |
|  |  | Reverse: GCCATGGCTAAGGTTTTCGT |  |
| ***FAS*** | NM_205155.2 | Forward: AGAGGCTTTGAAGCTCGGAC | 127 |
|  |  | Reverse: GGTGCCTGAATACTTGGGCT |  |
| ***ACC*** | NM_205505.1 | Forward: TTGTGGCACAGAAGAGGGAA | 161 |
|  |  | Reverse: GTTGGCACATGGAATGGCAG |  |
| ***SREBP-1c*** | XM_046927256.1 | Forward: GCCCTCTGTGCCTTTGTCTTC | 130 |
|  |  | Reverse: ACTCAGCCATGATGCTTCTTC |  |
| ***PPARγ*** | NM_001001460.1 | Forward: CCAGCGACATCGACCAGTTA | 182 |
|  |  | Reverse: TCCCATCCTTAAAGAGTTCA |  |
| ***ACOX1*** | XM_046929382.1 | Forward: GCCAGGTGGACTTGGAAAGA | 107 |
|  |  | Reverse: GCTGCCGTATAGGAACAATGAAG |  |
| ***FABP1*** | NM_204192.4 | Forward: AGAAGGCCAAGTGTATTGTTAACAT | 100 |
|  |  | Reverse: GTGATGGTGTCTCCGTTGAGTTC |  |
| ***NRF2*** | NM_205,117.1 | Forward: GATGTCACCCTGCCCTTAG | 216 |
|  |  | Reverse: CTGCCACCATGTTATTCC |  |
| ***HO-1*** | HM237181.1 | Forward: GGTCCCGAATGAATGCCCTTG | 137 |
|  |  | Reverse: ACCGTTCTCCTGGCTCTTGG |  |
| ***SOD*** | NM_205,064.1 | Forward: CCGGCTTGTCTGATGGAGAT | 125 |
|  |  | Reverse: TGCATCTTTTGGTCCACCGT |  |
| ***GSH-Px*** | NM_0,012,77853.1 | Forward: GACCAACCCGCAGTACATCA | 204 |
|  |  | Reverse: GAGGTGCGGGCTTTCCTTTA |  |

Note: *β-Actin* was used as an internal control. *PPARγ*, proliferator-activated receptors; *SREBP–1c*, Sterol regulatory element-binding protein 1c; *FAS*, fatty acid synthase; *FABP1*, Fatty Acid Binding Protein 1; *CPT1*, carnitine palmitoyltransferase 1; *ACOX1*, Acyl-CoA Oxidase 1; *SCD1*, Stearoyl-CoA Desaturase. *NRF2*, nuclear factor‑erythroid 2 related factor 2; *HO–1*, Heme Oxygenase-1; *GSH–Px*, glutathione peroxidase; *SOD*, Superoxide Dismutase; *CAT*, catalase.
